# Improving cancer detection through combinations of cancer and immune biomarkers: a modelling approach

**DOI:** 10.1186/s12967-018-1432-8

**Published:** 2018-03-20

**Authors:** Raluca Eftimie, Esraa Hassanein

**Affiliations:** 1 Division of Mathematics, University of Dundee, Dundee, DD1 4HN UK; 2 Biophysics Department, Faculty of Science, Cairo University, 12613 Giza, Egypt

**Keywords:** Ovarian cancer, Mathematical model, CA-125 biomarker, IL-7 biomarker, Cancer detection times

## Abstract

**Background:**

Early cancer diagnosis is one of the most important challenges of cancer research, since in many cancers it can lead to cure for patients with early stage diseases. For epithelial ovarian cancer (which is the leading cause of death among gynaecologic malignancies) the classical detection approach is based on measurements of CA-125 biomarker. However, the poor sensitivity and specificity of this biomarker impacts the detection of early-stage cancers.

**Methods:**

Here we use a computational approach to investigate the effect of combining multiple biomarkers for ovarian cancer (e.g., CA-125 and IL-7), to improve early cancer detection.

**Results:**

We show that this combined biomarkers approach could lead indeed to earlier cancer detection. However, the immune response (which influences the level of secreted IL-7 biomarker) plays an important role in improving and/or delaying cancer detection. Moreover, the detection level of IL-7 immune biomarker could be in a range that would not allow to distinguish between a healthy state and a cancerous state. In this case, the construction of solution diagrams in the space generated by the IL-7 and CA-125 biomarkers could allow us predict the long-term evolution of cancer biomarkers, thus allowing us to make predictions on cancer detection times.

**Conclusions:**

Combining cancer and immune biomarkers could improve cancer detection times, and any predictions that could be made (at least through the use of CA-125/IL-7 biomarkers) are patient specific.

## Background

Ovarian cancer is the most fatal of all gynecologic malignancies, since it is usually detected in the later stages when the 5-year survival is only between 37–44% [1, 2]. Until an effective treatment is found, early diagnosis (when the tumour can be treated more effectively) is the only option to improve patient outcome. In fact, early cancer detection could increase patients 5-year survival rates to even 90% [3–5].

As emphasised in various studies [3, 6], currently there are no non-invasive methods that could accurately detect early-stage ovarian cancers. The classical approach for ovarian cancer diagnosis involves the serum tumour biomarker CA-125 (carbohydrate antigen 125), which is elevated in the serum of most women with ovarian cancer [7]. However, despite its widespread clinical use, this biomarker does not seem to lead to significant increase in the survival rates of asymptomatic women [3]. Unfortunately, CA-125 lacks both sensitivity and specificity required for the efficient screening of ovarian cancers (where sensitivity is defined as the proportion of patients with ovarian cancer correctly identified by CA-125, while specificity is the proportion of patients without ovarian cancer correctly identified by CA-125 [8]).

To address this issue related to the CA-125 biomarker, the last 10–15 years have seen the development of various multimodal strategies that combine multiple diagnostic markers/tools [4, 9, 10]. For example, we note the combination of CA-125 with transvaginal sonography [11], with human epididymal secretory protein 4 (HE4) [12], or with mesothelin [13], to investigate the possibility of improving early cancer detection. An approach that has received particular attention in the past years focuses on the use of serum cytokine levels as diagnostic and prognostic markers in ovarian cancer [14, 15]. Many of these cytokines (e.g., IFN-$\gamma$, IL-2, IL-7, G-CSF, …) are produced by various hematopoietic and non-hematopoietic cell lines, and are involved in inflammation and immunity [14]. In addition, some cytokines (such as IL-6 and IL-8) seem to be produced also by ovarian cancer cells [14].

A cytokine that has been investigated in the context of ovarian cancer detection is IL-7 (interleukin 7) [14]. This cytokine, which is produced mainly by non-hematopoietic cells (e.g., epithelial cells in the thymus, prostatic epithelium and the intestine; see [16]) and by some immune cells (e.g., dendritic cells), is important for the development of B cells and T cells [17]. Moreover, IL-7 seems to have anti-tumour effects in tumours such as melanoma, prostate cancer or glioblastoma, and potential pro-tumour effects in bladder cancer by promoting cell invasion and migration [17]. Since high IL-7 serum levels have been detected in ovarian cancers [18–20], it has been suggested that IL-7 can be used in combination with CA-125 to distinguish between malignant and benign ovarian tumours [14]. Moreover [18], suggested that the elevated serum IL-7 is the result of host anti-tumour immunity.

The use of cytokines for cancer detection seems particularly relevant in the context of recent studies which emphasise more and more the importance of immune responses in the evolution of ovarian cancer and long-term patient survival [21–23]. Despite clinical observations that ovarian cancers can induce spontaneous anti-tumour immune responses [23], and that significant numbers of tumour-infiltrating lymphocytes have been found inside cancerous ovarian tissues (some immune cells being associated with improved overall survival [22]), the role of the immune system in response to ovarian cancer is still not fully understood. The poor outcome of this particular type of cancer is also the result of immune cells failing to control tumour growth, due to the recruitment inside the tumour environment of suppressive immune cells such as Tregs, or the NK cells failing to recognise tumour antigens [23]. However, there are not many studies in the literature that investigate the secretion of immune biomarkers (and their use for cancer detection) in the context of complex tumour–immune interactions.

Since mathematical approaches have been shown to be very useful on shedding light on the biological mechanisms behind various complex immune responses and on making further biological predictions [24], in this study we consider such an approach to investigate computationally the interactions between tumour cells and tumour-infiltrating lymphocytes (i.e., dendritic-cell-activated CD8$^{+}$ T cells), and the use of biomarkers associated with these different cells to improve cancer detection times.

Mathematical modelling and computational approaches have recently started being used to asses the detection level of cancer biomarkers, and they usually focus on one biomarker at a time, e.g., CA-125 [25, 26], SEAP [27], uPAR [28], or nanoparticles conjugated with protease-cleavable peptides [29]. While many of these models are deterministic (usually described by ordinary differential equation (ODE) models), the past 10 years have seen also a significant increase in the development of various stochastic network-based biomarker models for the diagnosis and investigation of different cancers [30–33]. Generally, these network models incorporate a large number of cancer-related proteins and networks of proteins, and use them to identify the most likely biomarkers for cancer detection. Hence, the two major approaches in the literature either (i) start with simple mathematical models of basic processes and then add more complexity, or (ii) start by considering the complexity of the system, and then try to deconvolute it to identify the most important processes. Throughout this study, we consider only the first approach.

Here, we start with a simple ODE model introduced in [25] for tumour growth and CA-125 secretion, and generalise it to investigate the use of two different biomarkers (CA-125 and IL-7) on the overall detection time. We chose to focus on these two biomarkers since [14] showed that IL-7 levels were strongly associated with ovarian cancer, and moreover a combination of IL-7 and CA-125 serum levels could accurately predict 69% of ovarian cancer patients. With the help of this new model, we show the importance of the heterogeneity in the immune response (which impacts the secretion level of IL-7) on the cancer detection times. Thus we show that by combining an immune biomarker with a cancer biomarker one could help improve tumour detection times in some patients, but also might delay tumour detection in other patients (depending on the level of anti-tumour immune response).

## Methods

We start modelling the tumour dynamics by considering (as in [25]) a mono-exponential model for early tumour growth (the density of tumour cells at time *t* being described by $N_{\text {T}}(t)$). To model an early anti-tumour immune response, we then couple the equation for the evolution of tumour cells $N_{\text {T}}(t)$ with an equation for the evolution of immune cells $N_{\text {I}}(t)$:

1a $$\begin{aligned} \frac{d N_{\text {T}}(t)}{dt}&=k_{gr}N_{\text {T}}(t)-d_{t}N_{\text {T}}(t)\frac{N_{\text {I}}(t)}{h_{i}+N_{\text {I}}(t)},\end{aligned}$$

1b $$\begin{aligned} \frac{d N_{\text {I}}(t)}{dt}&=a_{i}N_{\text {T}}(t)\Big (1-\frac{N_{\text {I}}(t)}{M}\Big )-d_{i}N_{\text {I}}(t). \end{aligned}$$

Here, $k_{gr}$ is the growth rate of the ovarian cancer cells, $d_{t}$ is the rate at which immune cells eliminate the detected tumour cells, $a_{i}$ is the activation/proliferation of immune cells in response to tumour antigens, $d_{i}$ is the natural half-life of immune cells, and *M* is the carrying capacity for the immune cells (since the body cannot support an extremely large number of activated immune cells, which would trigger a cytokine storm [34]). Note that we use the saturated term $N_{\text {I}}(t)/(h_{i}+N_{\text {I}}(t))$ to describe the immuno-modulating effect of ovarian cancer cells on the immune response, which leads to reduced anti-tumour immune responses (and subsequent cancer growth) [35]. Parameter $h_{i}$ is the half-saturation constant of immune cells that generate an anti-tumour immune response. Note that, for simplicity, here we assume that the generic immune cell population $N_{\text {I}}$ includes both antigen-presenting cells (e.g., dendritic cells) and anti-tumour effector cells (e.g., CD8$^{+}$ T cells) activated by these antigen-presenting cells upon detection of tumour antigens.

Next, we model the shedding of biomarkers by tumour and immune cells. For the CA-125 biomarker, we follow the approach in [25] and assume that the equation for the change in the amount of tumour plasma biomarkers, $B_{T}(t)$, which are shed by both tumour ($N_{\text {T}}$) and healthy ($N_{h}$) cells, is given as

2 $$\begin{aligned} \frac{dB_{\text {T}}(t)}{dt}=f_{ht}R_{ht}N_{h}+f_{t}R_{t}N_{\text {T}}(t)-k_{et}B_{\text {T}}(t). \end{aligned}$$

For simplicity, we assume that the number of healthy cells does not vary significantly in time, and thus we take $N_{h}=$ constant (i.e., the initial number of healthy cells). Parameters $f_{t}$ and $f_{ht}$ are the fractions of tumour biomarker entering the tumour and the healthy vasculatures, respectively. Parameters $R_{t}$ and $R_{ht}$ are the shedding rates of tumour biomarker from tumour and healthy cells, respectively. Finally, $k_{et}$ is the elimination rate of tumour biomarker from plasma.

In regard to the immune biomarkers, [18] showed that the ovarian carcinoma cells rarely express IL-7, with the authors hypothesising that the elevated level of IL-7 in the serum and ascites of ovarian cancer patients was mainly from the host immune cells. Because the IL-7 biomarker can be produced by the immune cells (e.g., dendritic cells which activate the CD8$^{+}$ T cells) and by the healthy non-hematopoietic cells ($N_{h}$), the equation for the change in the amount of the immune biomarker $B_{\text {I}}$ is given by

3 $$\begin{aligned} \frac{dB_{\text {I}}(t)}{dt}=f_{hi}R_{hi}N_{h}+f_{i}R_{i}N_{\text {I}}(t)-k_{ei}B_{\text {I}}(t). \end{aligned}$$

Here $f_{i}$ and $f_{hi}$ are the fractions of immune biomarkers (shed by immune and healthy cells) that enter the vasculatures, while $R_{i}$ and $R_{hi}$ are the shedding rates of the immune biomarker from immune and healthy cells. Finally, $k_{ei}$ is the elimination rate of immune biomarker from the plasma. As before, we assume that the population of healthy cells is constant: $N_{h}=$ constant.

**Fig. 1:**
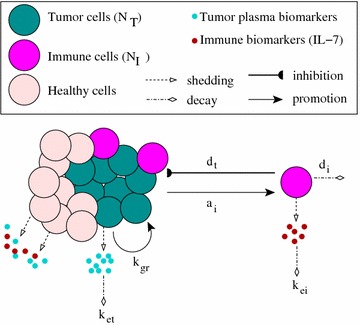
A schematic representation of the interactions between tumour cells and immune effector cells, as described by model (1)–(3)

These tumour–immune interactions and biomarker secretion dynamics are summarised in Fig. 1.

### Model parametrisation

Table 1 contains the baseline values and ranges for the parameters associated with the tumour biomarker (CA-125), as estimated by [25]. Table 2 contains the baseline values and ranges for the parameters associated with the immune response, which are estimated in the present study as follows:

1. *Immune cells turnover* [36] calculated the doubling time for CD8$^{+}$ T cells to about 8 h, and their half-life during the contraction phase to about 41 h. This translates into the following parameter values: $a_{i}=\ln (2.0)/8$ h = 2.0794/day, and $d_{i}=\ln (2.0)/41$ h = 0.4/day.
  However, since our variable $N_{\text {I}}$ accounts for a combination of effector CD8$^{+}$ T cells and antigen-presenting dendritic cells (which detect the tumour antigens, and further induce the activation and proliferation of CD8$^{+}$ T cells), we need to discuss also the turnover of dendritic cells—which can influence the overall dynamics of $N_{\text {I}}$ population in Eq. (1b), as suggested by [37]. In fact, [37] showed that different subsets of dendritic cells have different turnover rates, with the myeloid dendritic cells having a faster turnover than the plasmacytoid dendritic cells. However, the plasmacytoid dendritic cells have turnover rates similar to the CD8$^{+}$ T cells [37]. For this reason, throughout this study we consider the baseline $a_{i}$ and $d_{i}$ values defined above. Nevertheless, to describe the heterogeneity of $N_{\text {I}}$ population, throughout this study we will vary the rates $a_{i}$ and $d_{i}$ over the following parameter ranges: $a_{i}\in (0.2,3)$, and $d_{i}\in (0.2,0.6)$.
2. *IL-7 half life* The IL-7 half-life can range between 6.46 and 9.8 h, depending on the dose [38]. This half-life corresponds to an elimination rate between 2.575 and 1.6975/day. Throughout this study we choose a baseline value of $k_{ei}=2.14$/day.
3. *Immune production of IL-7* Since to our knowledge the fraction of IL-7 entering immune vasculature ($f_{i}$) has not been measured separately, we decided to focus on the overall shedding influx ($f_{i}R_{i}$). To this end, we note that [18] have estimated that the shedding influx of serum IL-7 in the healthy patients is 10.64 (pg/ml)/$10^{6}$cells/48 h, while the shedding influx of serum IL-7 in ovarian cancer patients is 32.49 (pg/ml)/$10^{6}$ cells/48 h. Moreover, the authors hypothesised that the elevated level of IL-7 in the serum and ascites of ovarian cancer patients is mainly from the host immune cells. Thus, we can assume that the shedding influx of IL-7 produced by the immune cells ($f_{i}R_{i}$) in ovarian cancer patients is equal to the difference between the shedding influx of IL-7 in the serum of ovarian cancer patients (32.49 pg/ml per $10^{6}$ cells per 48 h) and that in healthy patients (10.64 pg/ml per $10^{6}$ cells per 48 h), which equals 21.85 pg/ml per $10^{6}$ cells per 48 h. Thus, we use a baseline value of $f_{i}R_{i}=10.925\times 10^{-6}$ (pg/ml)/cell/day.
4. *Healthy cell production of IL-7* Assuming that the immune cells shed the immune biomarker (IL-7) as a response to tumour formation, then the production of IL-7 in healthy patients (in the absence of any immune responses) can be determined using the steady state mass ($B_{\text {I}}^{*}$) of the biomarker:
  $$\begin{aligned} f_{hi}R_{hi}N_{h}=k_{ei}B_{\text {I}}^{*} \end{aligned}$$
  The median concentration of IL-7 in healthy control subjects seems to vary between different studies. For example, [18] detected a median concentration of serum IL-7 in healthy patients of 10.64 pg/ml. In contrast, [14] detected a much lower serum median concentration of IL-7 in healthy patients: 2.9 pg/ml. In the following we consider these high and low serum IL-7 levels for the biomarker steady state $B_{\text {I}}^{*}$, and calculate the IL-7 influx as a result of production by the healthy cells:
  *High median serum IL-7 concentration* Knowing that the mean plasma volume in a 70-kg female patient is $V_{pl}=3150$ ml [25], we can obtain a baseline value for $B_{\text {I}}^{*}=10.64 \times V_{pl}=33516$ pg. For $k_{ei}=2.14$, we obtain a baseline influx value of $f_{hi}R_{hi}N_{h}=7.16\times 10^{4}$ pg/day. Moreover, since $k_{ei}\in (1.6975, 2.575/\text {day})$, we obtain that the IL-7 production by healthy cells can vary within the range $(5.69\times 10^{4}, 8.63\times 10^{4})$ pg/day.
  *Low median serum IL-7 concentration* In this case we obtain a baseline value for $B_{\text {I}}^{*}=2.9\times V_{pl}=9135$ pg. For $k_{ei}=2.14$, we obtain a baseline influx value of $f_{hi}R_{hi}N_{h}=1.9548\times 10^{4}$ pg/day. Moreover, since $k_{ei}\in (1.6975, 2.575/\text {day})$, we obtain that the IL-7 production by healthy cells can vary within the range $(1.55\times 10^{4}, 2.3522\times 10^{4})$ pg/day.
5. *Detection and cut-off limits for the immune biomarker assay* In [25] the authors considered two threshold values for the detection of CA-125 biomarkers: the detection limit $d_{CA125}$ (defined as the minimum concentration of biomarker detectable in plasma) and the cut-off limit $c_{CA125}$ (defined as the biomarker level that distinguishes a healthy from a disease state). For the detection of median serum IL-7 levels in ovarian cancer patients, [18] used a detection range of 0–2000 pg/ml with a sensitivity of 10 pg/ml. On the other hand, Mengus et al. [39] used a detection limit of 1 pg/ml for IL-7 in prostate cancer. Throughout this study, we use a baseline value of $d_{IL7}=1$ pg/ml. In regard to the cut-off limit of IL-7, [14] used a cut-off point of 3.8 pg/ml for IL-7 to distinguish between malignant and benign ovarian tumours (this corresponds to the case of low median IL-7 concentration). On the other hand, for the case of high median IL-7 concentration, [18] showed that the 25–75‰ for the serum IL-7 levels in patients with ovarian carcinoma are given by the range (13.56–54.60) pg/ml, the 25–75‰ in healthy control patients are given by (1.62–21.38) pg/ml, while the 25–75‰ in benign control patients are given by (0.07–25.73) pg/ml. Although the authors did not discuss a possible cut-off point, we can assume that this is between 13.5–25 pg/ml, with an average of 18 pg/ml. Thus, in this study we will investigate the effect of low and high cut-off points ($c_{IL7}=3.8$, $c_{IL7}=18$), corresponding to both low and high IL-7 serum levels.
6. *Tumor killing rate* We assume that the immune system fails to control tumour growth (due to limited anti-tumour response—see [19]) and so we use a tumour-killing baseline value of $d_{t}=10^{-6}$ cells/day. However, to test the sensitivity of the model to this parameter, we perform simulations for $d_{t}\in (10^{-12},10^{-3})$—see also Figs. 5 and 6.

**Table 1:** Description of parameter values involved in the CA-125 dynamics, as given in [25]

Parameter	Description (units)	Baseline value
$f_{ht}R_{ht}N_{h}$	Healthy cells shedding influx (U/day)	$4.56\times 10^{3}$
$f_{t}$	Fraction of tumour biomarker entering tumour vasculature	0.1
$R_{t}$	Biomarker shedding rate per tumour cell (U/day/cell)	$4.5\times 10^{-5}$
$N_{h}$	The constant level of healthy cells (which shed the CA-125 biomarker) (cell)	–
$k_{gr}$	Growth rate of tumour cell population (day$^{-1}$)	$5.78\times 10^{-3}$
$k_{et}$	Elimination rate of tumour biomarker from plasma (day$^{-1}$)	0.11
$c_{CA125}$	Cut-off limit of CA-125 assay (for healthy vs. disease states), when the biomarker is produced by both tumour and healthy cells (U/ml)	34.11
$d_{CA125}$	Detection limit of CA-125 assay (i.e., min concentration of biomarker detectable in plasma), when the biomarker is produced by the tumour cells alone (U/ml)	1.5
$V_{pl}$	Mean plasma volume in a 70-year female patient (ml)	3150
$D_{T}$	Tumour detection time (day)	To be determined

**Table 2:** Description of parameter values involved in the IL-7 dynamics

Parameter	Description (units)	Baseline values
$a_{i}$	CD8$^{+}$ T cells doubling time (day$^{-1}$)	2.0794
$d_{i}$	CD8$^{+}$ T cells half-life (day$^{-1}$)	0.4
$d_{t}$	Killing rate of tumour cells by immune effector cells (day$^{-1}$)	$10^{-6}$
$k_{ei}$	IL-7 half life (day$^{-1}$)	2.14
*M*	Carrying capacity of immune cells	$10^{9}$
$f_{i}R_{i}$	Influx of IL-7 secreted by immune cells, into the vasculature ((pg/ml)/cell/day)	$10.925\times 10^{-6}$
$f_{hi}R_{hi}N_{h}$	Healthy cells shedding influx of IL-7 (pg/day)	$1.9548\times 10^{4}$ (low shedding); or $7.1724\times 10^{4}$ (high shedding)
$c_{IL7}$	Cut-off limit of IL-7 assay (for healthy vs. disease states), when the biomarker is produced by both immune and healthy cells (pg/ml)	3.8 (low threshold) or 18 (high threshold)
$d_{IL7}$	Detection limit of IL-7 assay (i.e., min concentration of IL-7 detectable in plasma), when the biomarker is produced by the immune cells alone (pg/ml)	1.0
$V_{pl}$	Mean plasma volume in a 70-year female patient (ml)	3150
$D_{T}^{t}$	Tumour detection time based on the CA-125 biomarker (days)	To be determined
$D_{T}^{i}$	Tumour detection time based on the IL-7 biomarker (days)	To be determined

### Calculating tumour diameters

To calculate tumour diameters, we assume that a tumour with diameter $d=1$ cm contains approximately $10^{9}$ cells (as suggested in [40]). The volume of such a tumour, assumed to be perfectly spherical, is $V_{1}=(4/3)\pi (d/2)^{3}=\pi /6$ (for $d=1$ cm). The volume of a tumour with diameter $d_{x}$, which contains *x* cells, is $V_{d_{x}}=(4/3)\pi (d_{x}/2)^{3}$. Using the simple rule of three, we have $V_{d_{x}}=x\cdot V_{1}/10^{9}$, from which we can obtain the diameter $d_{x}$ of a tumour containing *x* cells: $d_{x}=(x/10^{9})^{1/3}$. We will use this formula in Fig. 9b, to calculate the tumour diameters at the biomarker detection times.

## Results

**Fig. 2:**
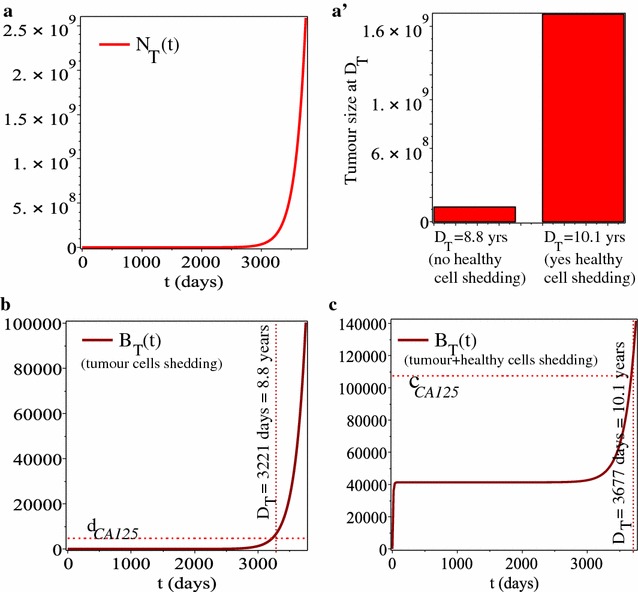
The dynamics of tumour cells and tumour biomarkers in the absence of any immune response (i.e., $N_{\text {I}}=B_{\text {I}}=0$), as investigated in [25]: **a** time-evolution of ovarian cancer cells; **a′** tumour size (cell numbers) at the detection times $D_{T}$, when the CA-125 biomarkers reach the detection thresholds (as shown in **b**, **c**); **b** dynamics of CA-125 biomarker, when we assume only tumour shedding. Horizontal line shows the biomarker detection threshold $d_{CA125}$. In this case, the tumour detection threshold is $D_{T}=8.8$ years; see also [25]; **c** dynamics of CA-125 biomarker, when we assume both tumour and healthy cells shedding. Horizontal line shows the biomarker cut-off threshold $c_{CA125}$. In this case, the tumour detection threshold is $D_{T}=10.1$ years; see also [25]. The parameter values for these simulations are given in Table 1

To compare our results for the combined use of two cancer biomarkers (i.e., CA-125 and IL-7) with the results in [25] for the use of only one cancer biomarker (CA-125), we start in Fig. 2 by showing the time-evolution of tumour cells and the CA-125 biomarker in the absence of any immune response or immune biomarker, under the assumptions that CA-125 can be produced by (b) tumour cells alone, or (c) tumour and healthy cells. (These results are obtained by considering only model (1a), (2), as in [25].) In panels (b) and (c) we also show the detection time ($D_{T}$) of the tumour, as calculated by determining the intersection point between the $B_{\text {T}}(t)$-curve and the CA-125 detection level $d_{CA125}$ (see panel b), or by determining the intersection point between the $B_{\text {T}}(t)$-curve and the CA-125 cut-off level $c_{CA125}$ (see panel c). Note that in panel (a′) we also show the size of the tumour at the detection times $D_{T}$ corresponding to the two cases shown in panels (b) and (c).

**Fig. 3:**
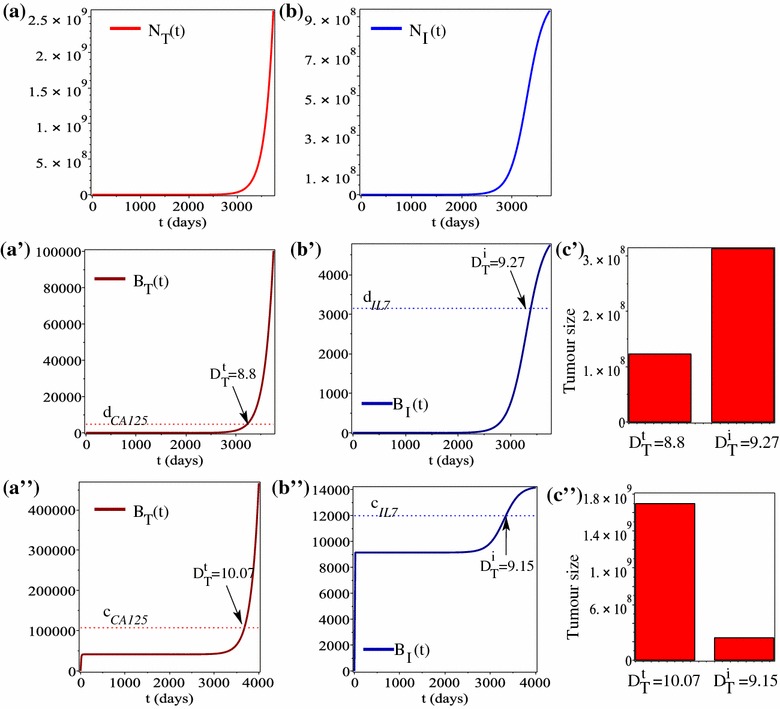
**a** Time-evolution of tumour cell population; **b** time-evolution of immune cell population; time-evolution of tumour biomarkers secreted by tumour cells alone (**a′**) or by tumour and healthy cells (**a″**); time-evolution of immune biomarkers secreted by immune cells alone (**b′**) or by immune and healthy cells (**b″**); **c′**, **c″** tumour size at the detection times $D_{T}^{t}$ and $D_{T}^{i}$ (corresponding to **a′**, **b′**, and **a″**, **b″** respectively). Parameter values for these simulations are given in Tables 1 and 2. The dotted horizontal lines in **a′**, **b′** show detection thresholds for CA-125 and IL-7 calculated by multiplying $d_{CA125}$ and $d_{IL7}$ with $V_{pl}$ = the mean plasma volume in a 70-kg female patient. The dotted horizontal lines in panels **a″**, **b″** show cut-off thresholds for CA-125 and IL-7 calculated by multiplying $c_{CA125}$ and $c_{IL7}$ with $V_{pl}$

In Fig. 3 we show the time evolution of tumour cells, immune cells, and tumour and immune biomarkers for the baseline parameter values listed in Table 2, corresponding to *low IL-7 serum baseline levels* (i.e., $f_{hi}R_{hi}N_{h}=1.9548\times 10^{4}$, $c_{IL7}=3.8$). We note that when the immune response is included, but we assume that IL-7 is produced only by the immune cells and CA-125 is produced only by tumour cells, then the tumour detection time based on CA-125 ($D_{T}^{t}=8.8$ years) is lower than the tumour detection time based on IL-7 ($D_{T}^{i}=9.27$ years); see panels a′, b′. However, when we assume that the tumour and immune biomarkers are produced also by the healthy cells in the environment, then $D_{T}^{t}=10.07>D_{T}^{i}=9.15$. Hence, the inclusion of the serum cytokine concentration in the detection tests for ovarian cancer, could lead to earlier detection of the tumour mass (i.e., almost 1 year earlier).

**Fig. 4:**
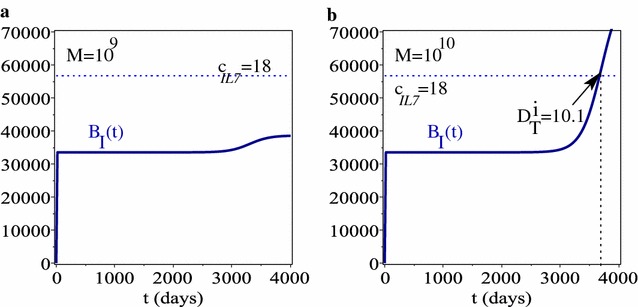
The time-evolution of immune biomarker concentration, for higher baseline IL-7 serum levels (i.e. $c_{IL7}=18$), as we increase the carrying capacity of immune cells (to allow the cells to reach higher numbers); **a** $M=10^{9}$; **b** $M=10^{10}$

In Fig. 4 we have also briefly investigated the effect of having *higher baseline serum IL-7 levels* (as in [18]). The results show that for lower immune carrying capacity values (i.e., $M=10^{9}$), the level of immune cells in the system does not produce enough IL-7 to be detected above the cut-off threshold of 18 pg/ml. (Since there are no changes in the evolution of $N_{\text {T}}(t)$, $N_{\text {I}}(t)$ or $B_{\text {T}}(t)$ compared to Fig. 3, we do not show here the dynamics of these variables.) To be able to detect between healthy and cancerous states it is necessary to either assume that there are more immune cells in the system (i.e., higher carrying capacity: $M=10^{10}$; see panel b) or higher secretion rate of IL-7 by the existent immune cells (i.e., higher $f_{i}R_{i}$—but we do not have any data to suggest these higher secretion values).

Since the heterogeneity of the immune response [41] can lead to small variations in the immune parameters, in the following we investigate how changes in the parameters associated with the immune response affect the tumour detection time. To this end, we focus only on the case of low baseline serum IL-7 (corresponding to the clinical data in [14]).

### Tumour detection times as we vary immune and tumour parameters

**Fig. 5:**
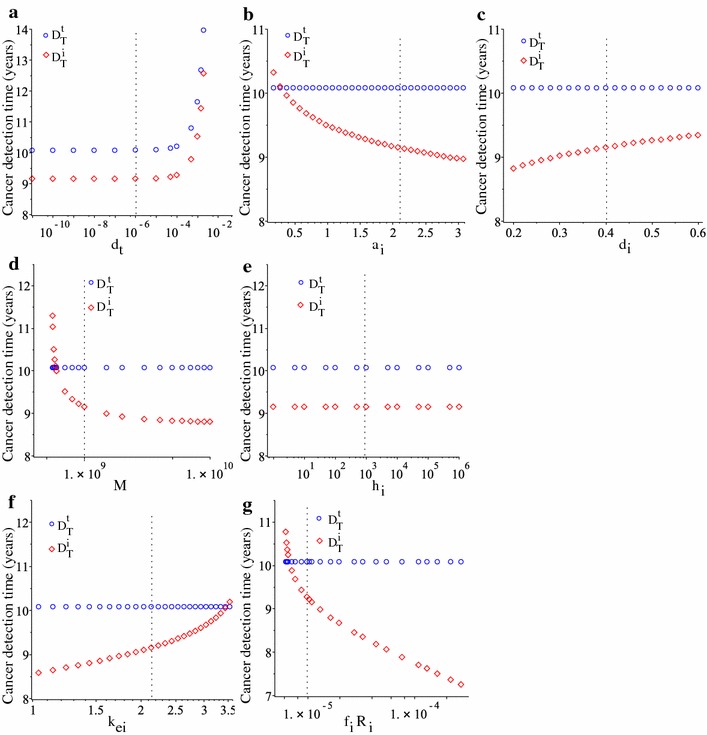
The effect of changing various immune-related parameters, namely **a** $d_{t}$, **b** $a_{i}$, **c** $d_{i}$, **d** *M*, **e** $h_{i}$, **f** $k_{ei}$, **g** $f_{i}R_{i}$, on the cancer detection times calculated based on the cut-off thresholds for the tumour biomarkers ($D_{T}^{t}$; blue circles) and the immune biomarkers ($D_{T}^{i}$; red diamonds). The vertical dashed lines denote the baseline parameter values, as listed in Table 2

Figure 5 shows the detection times $D_{T}^{t}$ and $D_{T}^{i}$ for the tumour and immune biomarkers, as we vary: (a) the tumour elimination rate $d_{t}$; (b) the immune activation/proliferation rate $a_{i}$; (c) the half-life of immune cells $d_{i}$; (d) the carrying capacity of immune cells *M*; (e) the half-saturation constant for the anti-tumour immune response $h_{i}$; (f) the degradation rate $k_{ei}$ of the immune biomarker; (g) the influx rate $f_{i}R_{i}$ of IL-7 that is secreted by immune cells. First, we note that variations in the half-saturation constant $h_{i}$ does not have any effect on the detection times (see Fig. 5e). Second, we note that variations in almost all other parameters can lead to an interchange between the time $D_{T}^{t}$ when the tumour biomarker reaches its cut-off threshold $c_{CA125}$ and the time $D_{T}^{i}$ the immune biomarker reaches its cut-off threshold $c_{IL7}$ (see Fig. 5b, d, f, g). For the baseline parameter values listed in Tables 1 and 2, we have $D_{T}^{i}<D_{T}^{t}$, suggesting that the immune response could be used to improve the overall tumour detection time. Some of these interchanges in the tumour/immune biomarkers detection times occur outside realistic parameter ranges (e.g., $k_{ei}\in (1.6975,2.575)$ as in [38], but in Fig. 5f we investigated the range $k_{ei}\in (1,3.5)$). However, they can inform us of possible dynamics in perturbed system (e.g., following immunotherapies for different diseases—other than cancer, which might affect also the immune response to cancer and the cancer detection times).

**Fig. 6:**
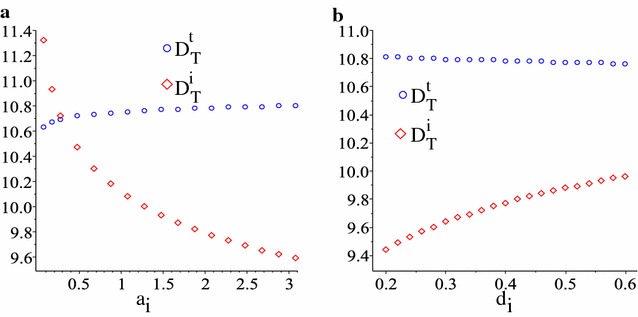
Tumour detection times, $D_{T}^{t}$ and $D_{T}^{i}$ for higher tumour lysis rate $d_{t}=5\times 10^{-4}$, as we vary two immune parameters: **a** $a_{i}$ and **b** $d_{i}$

We note in Fig. 5 that with the exception of changes in $d_{t}$, changes in all other immune-related parameters do not seem to affect the tumour detection time $D_{T}^{t}$ based on the CA-125 biomarker (such changes affect only $D_{T}^{i}$). The reason for this result is the very low tumour killing rate $d_{t}$ (which was assumed at a baseline value of $10^{-6}$/day—to explain the failure in the immune response to control tumour growth). Increasing this tumour killing rate could lead to small changes in $D_{T}^{t}$, as shown in Fig. 6 (for different $a_{i}$ and $d_{i}$ values). However, higher $d_{t}$ values also mean that tumours can be detected much later.

**Fig. 7:**
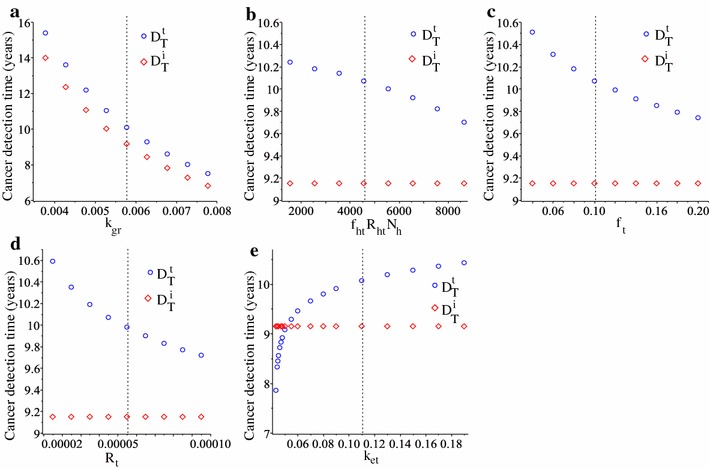
The effect of changing various tumour-related parameters, namely **a** $k_{gr}$, **b** $f_{ht}R_{ht}N_{h}$, **c** $f_{t}$, **d** $R_{t}$, **e** $k_{et}$, on the cancer detection times calculated based on the cut-off thresholds for the tumour biomarkers ($D_{T}^{i}$; blue circles) and the immune biomarkers ($D_{T}^{i}$; red diamonds). The vertical dashed lines denote the baseline parameter values, as listed in Table 1

Finally, in Fig. 7 we show the effects of varying tumour-related parameters on tumour detection times $D_{T}^{t}$ and $D_{T}^{i}$. We remark in panel (a) that changes in tumour proliferation rate $k_{gr}$ affect both tumour and immune biomarker levels, which subsequently affect the tumour detection times. In contrast, changes in all other tumour-related parameters affect only the tumour biomarkers.

### Tumour size at detection time

**Fig. 8:**
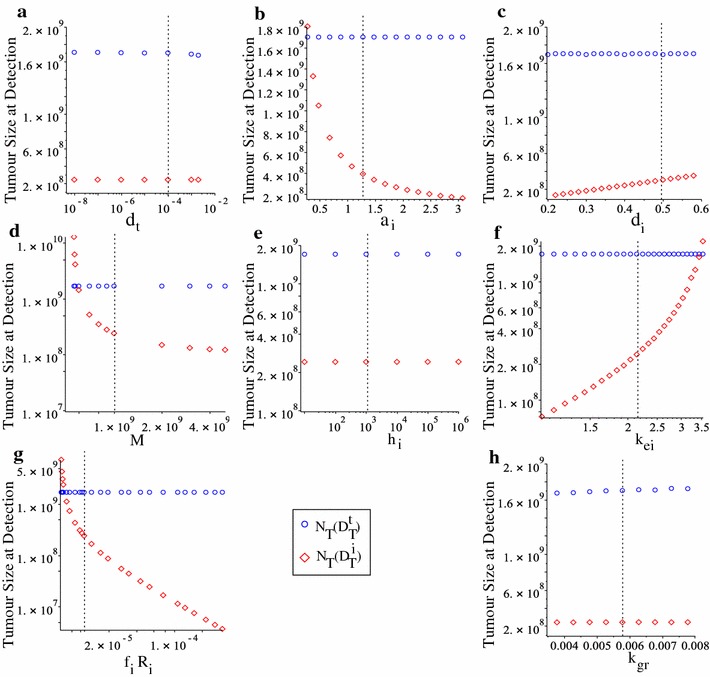
Tumour size at the detection times $D_{T}^{t}$ (blue circles) and $D_{T}^{i}$ (red diamonds), as we vary: **a**–**g** the immune parameters corresponding to a–g in Fig. 5; and **h** the tumour growth rate, corresponding to Fig. 7a

To investigate the size of the tumour at the detection time, in Fig. 8 we show $N_{\text {T}}(t)$ at times $t=D_{T}^{t}$ (blue circles) and $t=D_{T}^{i}$ (red diamonds). We note that for the majority of parameters the tumour size increases/decreases as we vary the parameters, in the same manner as the tumour detection times increase/decrease as we vary these parameters—see also Figs. 5 and 7. It is worth mentioning here that by varying some immune parameters, one could detect even very small tumours (e.g., tumours less than $10^{7}$ cells—see Fig. 8f, g).

There is one particular aspect that we need to emphasise in Fig. 8: changes in parameters $d_{t}$ and $k_{gr}$ do not lead to changes in tumour size at detection times (for either $D_{T}^{t}$ or $D_{T}^{i}$). This is in contrast with the effects of $d_{t}$ and $k_{gr}$ on the detection times (see Figs. 5a, 7a).

### Predictions of tumour evolution in the (IL-7, CA-125) phase space

**Fig. 9:**
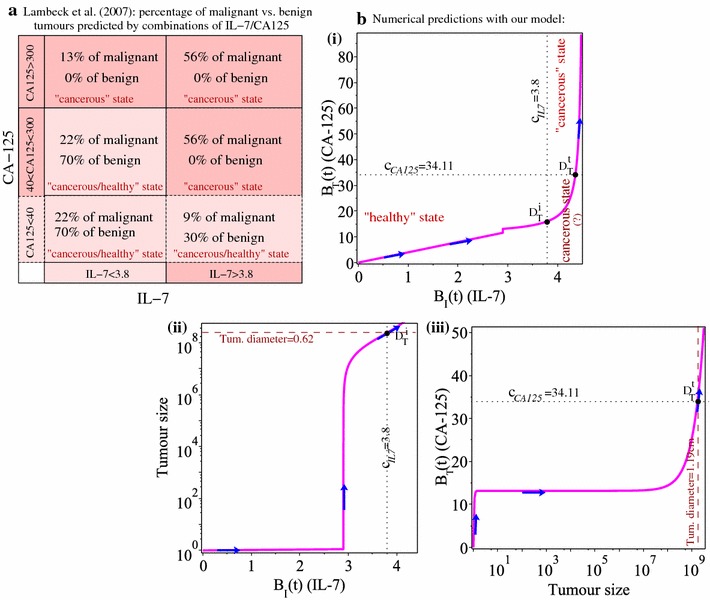
**a** Regions of malignant vs. benign tumour predictions in the (IL-7, CA-125) parameter space, as proposed in the clinical study by [14]. **b**(**i**) Regions of healthy vs. cancerous states in the (IL-7, CA-125) phase plane, as predicted by the numerical simulations; (**ii**) tumour size versus the immune biomarker (IL-7) level (the dotted vertical line shows the IL-7 detection threshold, while the dashed horizontal line shows tumour size at the detection time); (**iii**) tumour size versus the cancer biomarker (CA-125) level (the dotted horizontal line shows the CA-125 detection threshold, while the dashed vertical line shows tumour size at the detection time). The blue arrows on the solution curves show the increase in time

Finally, we discuss our results in the context of diagnosing ovarian cancer in the (IL-7, CA-125) variables space. We note that [14] investigated the levels of IL-7 and CA-125 that can be used to predict benign versus malignant ovarian tumours—see also Fig. 9a. In this figure we observe that for low CA-125 ($<40$), a reduced IL-7 level ($<3.8$) predicted 22% of malignant cases, while an increased IL-7 level ($>3.8$) predicted only 9% of malignant cases (despite the fact that malignant ovarian cancers are usually associated with higher IL-7 levels); see [14]. This discrepancy in the predictions could be the effect of low specificity and sensitivity of IL-7 [14], which is related to the multiple roles of IL-7 in the homeostasis of the immune system [42]. We will discuss this aspect in more detail in the next section.

Since our mathematical model does not distinguish between malignant and benign tumours, but rather the healthy from the cancerous states, in Fig. 9b(i) we graph $B_{\text {I}}(t)$ versus $B_{\text {T}}(t)$ and their cut-off thresholds. First we note that the “healthy” state is characterised by CA-125 $<15$ U/ml, which is consistent with clinical data on healthy control patients—see Table 2 in [14]. Second, for $t>D_{T}^{t}$ the tumour is detected through elevated tumour biomarkers and the “cancerous” state is characterised by CA-125 $>35$ U/ml. The region with CA-125 $<35$ and IL-7 $>3.8$ could be characterised by either a “healthy” or “cancerous” state, since up to 22% of cancerous patients have shown to exhibit lower CA-125 values [9] (and in fact, more recent CA-125 assays now accept a cut-off limit of 20 U/ml [2, 43]). For the parameter values used in this study (see Table 1), the dynamics of system (1)–(3) did not enter the space region characterised by $B_{\text {I}}(t)<3.8$ and $B_{\text {T}}(t)>35$; although [14] showed that there are clinical cases of malignant cancer in this region, as seen in panel (a). Such numerical results could be obtained for example for very low $a_{i}$, *M* or $f_{i}R_{i}$ values, when $D_{T}^{t}<D_{T}^{i}$ (as shown in Fig. 5).

Because the aim of this study is to investigate whether the addition of an immune biomarker could be used to improve early cancer detection, in Fig. 9b(ii), (iii) we graph tumour size versus the two biomarkers (IL-7 and CA-125), together with the tumour diameters at the detection times. (We explained how we calculated these diameters in “Methods” section.) Overall, these results suggest that by considering also immune biomarkers, we could detect tumours that have much smaller sizes: e.g., diameters of $\approx 0.62$ cm, as showed in Fig. 9b(ii). Moreover, lower tumours could be detected if different immune-related model parameters are varied (as shown in Figs. 5, 7a; and as supported by the heterogeneity of the immune response [41]). As we will discuss in more detail in the next section, this result could fall within the remits of the study in [44], which suggested that the detection sensitivity could increase to $\approx 80\%$ for tumours of $\approx 0.5$ cm diameters.

## Discussion

Improving the current methods of tumour detection is a critical question in tumour research. A few years ago, [25] proposed a simple mathematical model to predict tumour detection times based on the level of tumour biomarkers. Applying the model to ovarian tumours and the CA-125 biomarkers, the authors concluded that this type of tumour could grow undetected at least 8.8–10.1 years (from the moment the tumour starts forming). However, what the authors did not consider in their study is the immunogenicity of the tumours, especially since the epithelial ovarian cancers have been shown to be quite immunogenic [19, 22].

In this study we proposed a new mathematical model that described the interactions between tumour and immune cells, and the secretion of tumour biomarkers (CA-125) and immune biomarkers (IL-7) that could be both used to predict the presence of ovarian tumours.

Using this model, we showed that by combining tumour and immune biomarkers one can either increase or reduce the time for tumour detection, depending on whether the biomarkers are produced also by healthy cells. For example, assuming that CA-125 is produced only by cancer cells and IL-7 is produced only by immune cells lead to detection times $D_{T}^{t}=8.8$ years and $D_{T}^{i}=9.27$ years (see Fig. 3a′, b′). In contrast, assuming that CA-125 can be produced also by healthy cells lead to an increase in tumour biomarker detection time to $D_{T}^{t}=10.07$ years; see Fig. 3a″. However, assuming that IL-7 can be produced also by healthy cells lead to a decrease in immune biomarker detection time to $D_{T}^{i}=9.15$; see Fig. 3b″. This unexpected result is likely linked to the low cut-off value of IL-7 (as determined in [14]). Choosing higher cut-off IL-7 values (as in [18]) could delay the tumour detection time based on the immune biomarker; see Fig. 4. Hence, the results of this study depend strongly on data we used from the published literature.

Various clinical studies that investigated the relation between tumour size in early versus advanced cancers, have emphasised that early stage cancers grow locally to relatively large sizes (i.e., $<\,6$ cm) before they are detected and/or spread [45, 46]. Moreover, in the context of preclinical ovarian tumour sizes, [44] suggested that achieving a 50% sensitivity in tumour detection before tumours reached advanced stages would require the detection of tumours of 1.3 cm diameter, while a 80% detection sensitivity would require the detection of tumour of 0.5 cm diameter. Assuming as in [40], that tumours with diameters of 1 cm are formed of approximately $10^{9}$ cells (see Fig. 1B in [40]), then a tumour of 0.5 cm diameter would contain approximately $1.25\times 10^{8}$ cells (see also the calculation of tumour diameters at the end of “Methods” section). Note that the tumour sizes calculated in Fig. 3c′, c″, in the context of immune biomarker detection, contain between $1\times 10^{8} - 5\times 10^{8}$ cells (corresponding to tumours with diameters between 0.464–0.79 cm). Hence, we suggest that the inclusion of immune biomarkers could increase also the detection sensitivity of ovarian cancers. Nevertheless, for a more detailed investigation of tumour detection sensitivity, we would need to fit model (1)–(3) to patient data (an exercise that would also give us better information on the variability of different immune-related parameters).

With the help of this mathematical model we also investigated the dependance of tumour detection times on the parameters controlling the immune response. In Fig. 5 we showed that changes in the majority of immune-related parameters (e.g., $d_{t}$, $a_{i}$, *M*, $k_{ei}$, $f_{i}R_{i}$)—which could be the result of the heterogeneity in immune responses [41]—have significant impacts on the time when ovarian tumours are detected, and on the size of the tumour at detection time as shown in Fig. 8b–g. Also changes in tumour-related parameters impact the time when the tumour is detected; see Fig. 7.

In regard to tumour size at detection, we have seen that two parameters, $d_{t}$ and $k_{gr}$, do not seem to have any effect on tumour size when the tumour is detected. However, these two parameters do impact both biomarker detection times $D_{T}^{t}$ and $D_{T}^{i}$ (see Figs. 5a, 7a). This suggests that the change in these parameters (e.g., an increase in $k_{gr}$ in Fig. 7a) leads to a change in detection times (e.g., a decrease in both $D_{T}^{t}$ and $D_{T}^{i}$) which is opposite but of the same magnitude as the change in tumour size (e.g., increase in tumour size), so that there is no overall variation in tumour size at the new detection times. The interesting aspect is that this particular behaviour can be found only in the two parameters that affect both $D_{T}^{t}$ and $D_{T}^{i}$ at the same time (all other immune-related parameters affect only $D_{T}^{i}$, and all tumour-related parameters affect only $D_{T}^{t}$). We believe that this is the effect of linear tumour growth and decay [although the decay term in Eq. (1a) has the tumour variable $N_{T}$ multiplied by a saturated term which depends on the immune response $N_{I}$; but for large $N_{\text {I}}$ this saturated term behaves as a constant].

Finally, we showed in Fig. 9b that by creating a ($B_{\text {I}},B_{\text {T}}$) phase space diagram (corresponding to model dynamics for patient-specific immune and cancer parameters), we could predict the evolution of the tumour detection based on the solution trajectory crossing the cut-off limits $c_{CA125}$ and $c_{IL7}$. We note here that the levels of CA-125 and IL-7 biomarkers could be in a space region that would not allow to distinguish between a healthy from a cancerous state (as observed clinically by [14] for IL-7 $>3.8$ and CA-125 $<40$, in the case of benign vs. malignant tumour; see also Fig. 9a). However, the construction of a ($B_{\text {I}},B_{\text {T}}$) diagram—for patient-specific parameters—could allow us to make predictions regarding the long-term evolution of the biomarker levels in individual patients.

Given the simplicity of the mathematical model (1)–(3), this study could not be applied to make predictions regarding benign versus malignant tumours (and thus we discuss our results in terms of “healthy” versus “cancerous” states). Future work on this topic would see a generalisation of model (1)–(3) to include also a benign tumour—to test the predictions of this particular model for the detection of either malignant or benign tumours (based on the CA-125/IL-7 classification of [14]). Another restriction of this study is related to the multifaceted role of IL-7: on boosting the immune response [16, 18], and on the possibility of IL-7 to act as a growth factor for ovarian cancer cells [14]. In addition, the CA-125 marker seems to have a (less understood) role in the immune response, for example by inhibiting the cytotoxic responses of NK cells [47], which requires further investigation.

Finally, given the complexity of the ovarian cancer evolution (which is evident from the heterogeneity of these cancers [48–50]), one could say that such simple mathematical models could be useless for understanding the disease. However, the goal of these simple models is not to reproduce all the details of the disease. Rather, these models could be used to identify common biological characteristics that can be further investigated experimentally [24]. The model introduced in this study (which focused on two very general cancer and immune biomarkers) can be easily generalised (see the discussion in [24]) to incorporate more complex aspects of ovarian cancer evolution: different cancer clone populations with different growth rates and different surface markers [51], or changes in the core molecules of different canonical pathways (such as PTEN, notch, PI3K/AKT, etc.) [51], or multiple cancer/immune biomarkers that might be associated also with different subtypes of ovarian carcinoma [52]. Moreover, the interaction rates between the different components of the system, as well as the cells proliferation/death rates and the biomarker secretion rates, could be made probabilistic. Such generalisations can transform the simple differential equations model (1)–(3) into more complex deterministic and stochastic network models [53, 54], which can include more biological realism. In the future, we will consider also a generalisation of model (1)–(3) to investigate multiple biomarker detection in the context of heterogeneous ovarian cancers. The relevant cancer biomarkers to be used in this model could be identified with the help of network models (see, for example, [31]).

## Conclusions

Using a new mathematical model, we investigated the dynamics of cancer-immune interactions and biomarker secretion by both immune and cancer cells, and showed that variations in immune-related parameters can affect the tumour detection times. Since in addition to tumour immune modulation there are many other factors that affect the level of immune responses—e.g., unrelated infections which lead to an active immune response and increased secretion of IL-7 (as in HIV infections [55] or *A. benhamiae* infections [56]), making accurate predictions regarding tumour detection times (based on both tumour and immune biomarkers) might depend significantly on the status of immune response in each patient. This idea is consistent with many other recent studies that discussed the promises and pitfalls of immune biomarkers as personalised medicines [57, 58]. However, the mathematical framework presented here could take the research one step further by incorporating patient-specific parameters (e.g., different levels of immune activation or biomarker secretion), and through the use of mathematical tools (e.g., sensitivity analysis or bifurcation diagrams) one could get a better understanding of the time-evolution of the tumour–immune-biomarker system.

To be able to use this model for predictive clinical purposes, one needs to have accurate data (at multiple time points) on the level of immune responses, as well as the levels of tumour and immune biomarkers and their cut-off values. We showed in Fig. 4 that changes in the cut-off values of IL-7 (corresponding to the values published in different clinical studies; see [14] vs. [18]) leads to different predictions regarding the detection of cancer based on immune biomarkers. However, once this data is available, such a mathematical model could be applied to individual patients.

We conclude by mentioning that since high levels of CA-125 are not exclusive to ovarian cancers (being found also in patients with breast, lung or gastrointestinal cancers [2]), and since IL-7 is a cytokine that characterises an active immune response, we hypothesise that a combined use of CA-125 and IL-7 could be employed to detect possible early signs of other types of cancers. In this case, other tumour/immune biomarkers need to be used in combination with CA-125/IL-7, to increase the specificity towards ovarian cancers [9], or towards other cancers.

## Abbreviations

ODE: ordinary differential equation
CA-125: carbohydrate antigen 125
IL-7: interleukin 7
HE4: human epididymal secretory protein 4
G-CSF: granulocyte-colony stimulating factor
uPAR: urokinase-type plasminogen activator receptor
SEAP: secreted embryonic alkaline phosphatase
